# Sodium-Glucose Cotransporter-2 Inhibitors Improve Cardiovascular Dysfunction in Type 2 Diabetic East Asians

**DOI:** 10.3390/metabo11110794

**Published:** 2021-11-21

**Authors:** Muhammad Afzal, Fahad A. Al-Abbasi, Muhammad Shahid Nadeem, Sultan Alshehri, Mohammed M. Ghoneim, Syed Sarim Imam, Waleed Hassan Almalki, Imran Kazmi

**Affiliations:** 1 Department of Pharmacology, College of Pharmacy, Jouf University, Sakaka 72341, Saudi Arabia; afzalgufran@ju.edu.sa; 2 Department of Biochemistry, Faculty of Science, King Abdulaziz University, Jeddah 21589, Saudi Arabia; fabbasi@kau.edu.sa (F.A.A.-A.); mhalim@kau.edu.sa (M.S.N.); 3 Department of Pharmaceutics, College of Pharmacy, King Saud University, Riyadh 11451, Saudi Arabia; salshehri1@ksu.edu.sa (S.A.); simam@ksu.edu.sa (S.S.I.); 4 Department of Pharmacy Practice, College of Pharmacy, AlMaarefa University, Ad Diriyah 13713, Saudi Arabia; mghoneim@mcst.edu.sa; 5 Department of Pharmacology, College of Pharmacy, Umm Al-Qura University, Makkah 21955, Saudi Arabia; Whmalki@uqu.edu.sa

**Keywords:** cardioprotection, East Asians, diabetes mellitus, SGLT2 inhibitors, insulin independent

## Abstract

In East Asians, the incidence of type 2 DM (T2DM) has increased as a result of major alterations in life. Cardiovascular problems are more likely in those with T2DM. Sodium-glucose cotransporter-2 (SGLT2) inhibitors are novel insulin-independent antihyperglycemic drugs that limit renal glucose reabsorption and thereby improve glycemic control. They are used alone or in combination with insulin and other antihyperglycemic medications to treat diabetes, and they are also helpful in protecting against the progression of complications. This review has evaluated the available evidence not only on the efficacy of SGLT2 inhibitors in T2DM, but also on their favourable cardiovascular events in East Asians. DM is an independent risk factor for cardiovascular diseases. As a result, in addition to glycemic control in diabetes management, the therapeutic goal in East Asian diabetic patients should be to improve adverse cardiovascular outcomes. Besides establishing antidiabetic effects, several studies have reported cardioprotective benefits of SGLT2 inhibitors via numerous pathways. SGLT2 inhibitors show promising antidiabetic drugs with potential cardiovascular advantages, given that a high number of diabetic patients in East Asia have co-existing cardiovascular disorders. Despite significant positive results in favour of SGLT2, more research is needed to determine how SGLT2 inhibitors exert these impressive cardiovascular effects.

## 1. Introduction

Diabetes mellitus (DM) is a complex metabolic disorder with a serious global impact on community health concerns in the 21st century [1]. Diabetes is becoming more common worldwide, with Asia leading the way [2]. The rise in the occurrence of type 2 DM (T2DM) can be attributed to ageing, rapid urbanization, and some environmental and genetic factors in Asia [3]. Undiagnosed diabetes and poor glucose tolerance are also significant contributors to Asia’s high prevalence of diabetes [4]. Several distinguishing elements of the Asian region can be used to extrapolate the causes of this significant rise in diabetes incidence. The prevalence and long-term management of diabetic complications have increased alarmingly, imposing a massive economic hardship on countries and healthcare systems. Diabetes consumes approximately 5% to 20% of total health spending in many countries, posing a barrier to long-term economic growth [5].

## 2. Feature Affects the High Occurrence of Diabetes in East Asians

**Figure 1:**
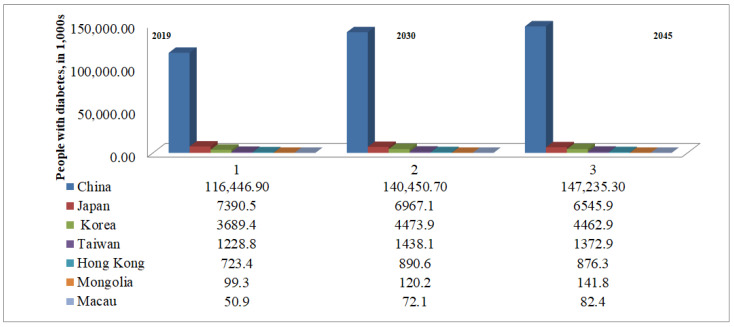
Comparison of information about diabetes between East Asian countries.

The International Diabetes Federation (IDF) has reported the national and international incidence of diabetes since 2000. In the IDF Diabetes Atlas, 9th edition, an estimated approximately 436 million individuals are affected worldwide, with a considerable proportion being East Asians. By 2030, the global load is predicted to reach 578 million people, rising to seven hundred million by 2045 [1]. Furthermore, according to the most recent IDF Diabetes Atlas 2019, 9th edition, approximately 116.4 million Chinese people have diabetes, making China the country with the highest number of cases in the world [1]. Figure 1 depicts a comparison of East Asian countries’ diabetes statistics (Diabetes Atlas, 2019). The factors affecting the high frequency of diabetes in Asian people with a typical ethnic background include lifestyle changes, metabolic syndrome, and an increase in the occurrence of obesity and T2DM. In many East Asian countries, urbanization, industrialization, and internal rural-to-urban migration are rapidly occurring, resulting in a number of negative consequences, including decreased exercise, a shift in diet to high calorie and significant increases in BMI (body mass index) and adiposity in the upper body [6,7,8]. Additionally, as a result of socioeconomic advancement, the proportion of people suffering from diseases caused by daily life modifications, i.e., DM, obesity, and cardiac disease, has increased. In terms of genetic and ecological risk factors for DM and cardiovascular disease, Asian populations have several important characteristics. Growing scientific data suggests that no one therapy prescription can be made for the entire world’s population.

### Genetic Factors

Type 2 diabetes is caused by complex interactions between multiple genetic susceptibility factors, as well as environmental and behavioural factors. Several diabetes genes have recently been revealed in Asians according to genome-wide association studies [9,10]. Several type 2 diabetes mellitus (T2DM) susceptibility loci have been identified in or near GLIS3, PEPD, FITM2-R3HDML-HNF4A, KCNK16, MAEA, GCC1-PAX4, PSMD6, and ZFAND [11]. The majority of these polymorphisms are thought to have an impact on T2DM risk by altering insulin secretion. GLIS3 has been linked to fasting glucose levels and has been linked to pancreatic beta cell development and insulin gene expression. TCF7L2 regulates the gene expression of relevant receptors, which modulates beta-cell responses to GLP-1 and GIP. Aside from nuclear DNA variants, mitochondrial DNA (mtDNA) polymorphisms/mutations, such as the mtDNA A3243G mutation and the mtDNA T16189C polymorphism, are also essential in determining diabetes risk [12].

## 3. Risk Factors Associated with Incidence of T2DM and Complications

Several risk factors lead to the incidence of diabetes and complications, including BMI (body mass index), extra visceral fat, diabetes onset at an early age, significant historical changes over the last decade, cardiovascular disease (CVD), insufficient β-cell response to counter insulin resistance, high alcohol intake, sedentary lifestyle, obesity or being overweight, high rate of childhood obesity, gestational diabetes, and increased inflammatory markers [13].

The previous review of Asian diabetes epidemiology established that untreated diabetes and impaired blood glucose tolerance reflect the rising incidence of DM in various East Asian countries [2]. Furthermore, the rise in gestational diabetes mellitus brings with it additional issues, such as DM’s risk in females and longstanding repercussions on the newborn or progeny. The high frequency and risk factors of gestational DM have been found in a systematic review study, which may be attributable to the grow in mother age and obesity [14]. One of the most striking observations in East Asians is the prevalence of T2DM at a body mass index [15]. According to the large cohort, having an elevated BMI was related to an augmented risk of DM due to insulin resistance and deteriorating β-cell function [16]. Higher fatty acid inflow into the liver, altered adipokine synthesis, hepatic steatosis, and hepatic glucose resistance are all possible consequences of increased visceral adiposity [17]. Ectopic fat accumulation in non-adipocyte tissue leads to cellular dysfunction and death, including inflammation and finally apoptosis. As a result, lipotoxicity in β-cell may thus reduce and dysfunctional secretion cause T2DM [4]. As highlighted earlier, increased insulin resistance due to visceral adiposity tissue can disrupt the balance, resulting in minimised insulin-production ability in East Asians [18]. Because of genetic abnormalities affecting β-cell mass and insulin production, East Asians have an increased chance of acquiring diabetes. Multiple unique T2DM loci have been discovered in East Asian investigations, with many of these variations projected to alter T2DM risk via influencing insulin secretion [19]. They could play a role in Asians’ lower insulin production activity. The exact mechanisms underlying these and their subsequent associations remain unclear. Both thirty-five-genotype and -phenotype hypotheses appear to have a role in the spread of DM among Asians, which is marked by an early age of onset, a proclivity for β-cell malfunction, and abdominal obesity [20]. T2DM is a predisposition to expand diabetes at an early age in the East Asian population. Due to genetic susceptibility, early development of T2DM has elevated the risk of micro- and macrovascular issues, a greater proclivity for β-cell dysfunction, and long-term disease duration [21]. The rising popularity of weight gain, particularly among young males, is a major contributor to the onset of T2DM. Even though not all obese people get DM, it is obvious that they are at a higher risk. Elevated levels of inflammatory indicators have been linked to the onset and development of cardiac events and T2DM [22]. The association between elevated markers of inflammation and constant cardio-metabolic diseases has been studied in epidemiology and clinical investigations [23]. Among some proinflammatory cytokines discovered within interleukin 1 (IL-1), islets cells seem to play an important role in the initiation and propagation of β-cell inflammation and dysfunction [24]. Lascar et al. proposed a possible association between young-onset T2DM and an increased pro-inflammatory pathway [25]. In addition, young-onset T2DM faces some challenges in diabetes management, such as the complexity of drug treatment, acceptance of insulin therapy, adherence to blood glucose monitoring, social stigma, and job stress. As a result, antihyperglycemic medications lower blood glucose levels, have a longer glycemic effect, and are easier to administer [26].

## 4. SGLT2 Inhibitors in T2DM Cure in Asians

East Asians have also increased the continued burden of diabetes [27]. Insulin-independent SGLT2 (sodium-glucose cotransporter) inhibitors are a type of antidiabetic drug. Sodium-glucose cotransporters (SGLT) are two distinct types of active cotransporters (types 1 and 2) that are primarily found in the brush border of the S2 and S3 segments of the proximal renal tubules as well as in the intestines. By inhibiting SGLT2, these drugs prevent glucose and salt from being reabsorbed in the proximal renal tubule, resulting in increased sodium and glucose excretion in the urine. Inhibitors of SGLT2 provide an antihyperglycemic action that is independent of insulin but reliant on renal function and plasma insulin level, the impact becoming less active when glucose falls below the normal physiological range [28]. The American Diabetes Association and the European Diabetes Association both recommend SGLT2 inhibitors as first- or second-line DM treatment [29]. The most common side effect of SGLT2 inhibitors appears to be genito-urinary genital infections, which have been shown to rise up to fourfold in clinical trials. Insulin-independent secretion mechanisms enable SGLT2 inhibitors to be used alone or in combination with insulin and other antihyperglycemic drugs. Additionally, it is expected to be useful in East Asian T2DM patients, especially considering the reduced insulin secretory capacity in Asians. SGLT2 inhibitors have been shown to improve insulin susceptibility and β-cells and enhance cardiac and renal outcomes in people with T2DM, according to recent research [29,30,31]. The SGLT2 inhibitor was found to lower systolic and diastolic pressure in both elevated-pressure cases and non-hypertensive DM [32]. By inhibiting SGLT2, a minimum affinity and high capacity transporter, glucose reabsorption in the proximal tubule is mediated [33]. Previous research suggested that SGLT2 and overexpression of glucose transport 2 enhanced renal glucose reabsorption in T2DM patients [4]. Evidence was proposed that SGLT2 inhibitors lowered the renal glucose excretion threshold. Furthermore, SGLT2 inhibitor-induced glycosuria enhances insulin and β-cell function, resulting in lower blood glucose levels [34]. Succeeding studies in global genetic knockout mice showed that SGLT2 inhibitors were linked to decreased plasma insulin level, higher insulin susceptibility, and improved β-cell activity, all of which supported this concept [35]. SGLT2 inhibitors increased glucagon release from α-cells in the pancreatic islet, which increased endogenous glucose synthesis. In addition to their effects on glucose homeostasis, SGLT2 inhibitors have been found to lower CVD risk [36]. SGLT2 inhibitors consist of different mechanisms of glucose control effect with reduced insulin resistance and sustained long-term glycemic control; insulin-independent action ensures longer control, systolic blood pressure, BMI, albuminuria, visceral fat, uric acid, plasma volume, arterial stiffness, inflammation, oxidative stress, sympathetic nervous system, and major adverse cardiac events [33,37,38,39,40]. In the United States and European Union, the Food and Drug Administration has permitted dapagliflozin, canagliflozin, and empagliflozin (SGLT2 selective inhibitors) [41,42,43]. Key studies of SGLT2 inhibitors, with either mono or dual therapy, in East Asians with T2DM are listed in Table 1. When compared to other second-line medications and others, SGLT2 inhibitors have proved to have greater blood glucose-lowering effects. In East Asians, the prevalence of low insulin caused by SGLT2 inhibitors were moderately lower and well tolerated [44]. In East Asia, multiple innovative scientific studies have shown the efficacy of SGLT2 inhibitors in lowering sugar levels. SGLT2 inhibitors were shown to lower HbA1c and insulin dosage in diabetes mellitus patients without a significant risk of hypoglycemia by Yang et al., suggesting that insulin resistance is improving [45]. In individuals with T2DM, clinical studies using the most advanced SGLT2 inhibitors, dapagliflozin and canagliflozin, have shown therapeutic improvements in terms of glycemic control, plasma glucose level, and reduced weight gain. HbA1c and bodyweight reductions have shown an additive effect of combining SGLT2 inhibitors with metformin [46]. According to studies, add-on SGLT2 inhibitors provided a better glucose-lowering impact than switch treatment [47,48]. A previous study in Taiwan reported a similar finding: those who switched from a DPP-4 inhibitor to an SGLT2 inhibitor experienced significant improvements, whereas those who remained on a DPP-4 inhibitor did not [49,50]. In Japanese patients, luseogliflozin combined with liraglutide improves glycemic control and leads to weight loss [51]. SGLT2 inhibitors are drugs that prevent the enzyme SGLT2 from functioning properly. Tofogliflozin may help patients recover from impaired β-cell activity if their insulin secretion capacity is preserved to some extent, according to a study of patients with elevated insulin stages at start-up [52]. In East Asian patients with T2DM, empagliflozin was found to be a well-tolerated and safe choice in placebo-controlled trials by pooled analysis [53].

## 5. Cardiovascular Axis of SGLT2 Inhibitors

**Figure 2:**
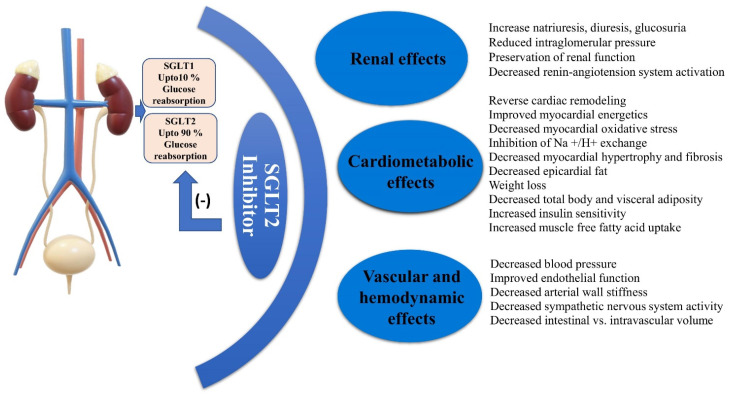
The proposed cardiovascular axis of SGLT2 inhibitor via several pleiotropic mechanisms.

In contrast to many other anti-diabetic agents, SGLT2 inhibition represents a promising approach to treating diabetes (Figure 2). SGLT2 inhibitors have been explored in a number of cardiac trials in patients with T2DM, with SGLT2 inhibitors lowering cardiac events [61,62,63]. In type 2 diabetic patients with a high CV risk or a history of severe CVD or advanced renal illness, SGLT2 inhibitors significantly prevent major adverse cardiovascular events, CV death, heart failure, and renal outcomes [64,65]. In individuals with T2DM, SGLT2 inhibitors improve cardiac performance indirectly by increasing natriuresis and diuresis, which decrease interstitial and plasma fluid quantities, dropping cardiac preload [66]. A decrease in stable cardiac rate was also linked to improvements in adipose tissue insulin resistance [67]. Enhanced cardiac fitness and the combination of SGLT2 inhibitors and loop diuretics was explored in people with T2DM and congestive heart failure [68,69]. Furthermore, SGLT2 inhibitors have been shown to create a condition of “fasting mimicry”, which stimulates the adenosine monophosphate-activated protein kinase and enzymes sirtuin 1, whose favourable anti-inflammatory actions may aid in heart function improvement [70]. SGLT2 inhibitors lower arterial blood pressure without increasing heart rate significantly, suggesting a link with a decrease in the sympathetic nervous system [71]. SGLT2 inhibitors show favourable effects on the sympathetic nervous systems function’s circadian rhythm, which may contribute to SGLT2 inhibitors’ clinical ability to improve blood pressure profiles and might produce good cardiac outcomes [72]. It has also been proposed that SGLT2 inhibitors can induce vasodilation by triggering the voltage-gated potassium channels and protein kinase G [73]. The direct vascular effects of SGLT2 inhibition, in addition to the natriuresis effects of SGLT2 inhibition, may play a role in the favourable hemodynamic effects described with SGLT2 inhibition. SGLT2 inhibitors natriuretic effect may contribute to cardiorenal benefits by inhibiting the myocardial sodium–proton exchanger, which has been shown to reduce cardiac hypertrophy and heart failure [74]. SGLT2 inhibitors raise glucagon levels, which have inotropic and chronotropic effects on the heart [75]. SGLT2 inhibitors have the potential to alter electrophysiology in the heart. A previous study found that using SGLT2 inhibitors decreases arterial stiffness and improves endothelial dysfunction [76]. Moreover, attenuation in endothelial dysfunction and arterial stiffness by reducing oxidative stress has also been reported, bringing potential benefits in vascular diseases. Diabetes, both type 1 and type 2 DM, empagliflozin improves arterial stiffness and vascular resistance [77]. Furthermore, glucosuria caused by SGLT-2 inhibition causes a series of metabolic changes that are likely to decrease fibrosis, inflammation and plaque formation, all of which have potential cardiac effects [78]. According to a previous study, dapagliflozin can reduce myocardial fibrosis and cardiac remodelling through modulating macrophage morphologies [79]. Inhibition of SGLT2 protects the cardiac against ischemia/reperfusion damage [80]. This beneficial effect is linked to a decline in calmodulin kinase II activity, which leads to increased Ca^2+^ flux and contractility in the sarcoplasmic reticulum. SGLT2 inhibition reduces epicardial fat mass and levels of bioactive molecules such as tumour necrosis factor-a and plasminogen activator inhibitor-1 in diabetic patients with CVD [81]. This may help to reduce the adverse remodelling of the failing heart. Empagliflozin considerably lowers cardiac interstitial fibrosis, pericoronary arterial fibrosis, arterial thickness, cardiac interstitial macrophage infiltration, and cardiac superoxide levels, according to Lin et al. [82]. Higher cardiac ketone oxidation mediated by empagliflozin gives an extra source of fuel for the heart in diabetic cardiomyopathic mice, which is allied with enhanced cardiac activity [83].

**Figure 3:**
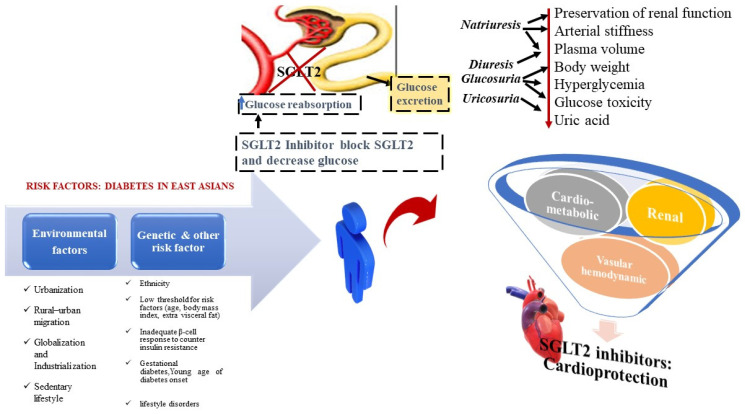
Effects of SGLT2 inhibitors on Type 2 diabetes mellitus and cardiovascular system.

Dapagliflozin reduces heart failure hospitalisation and cardiac events in DM patients, according to a large-scale randomised controlled trial and real-world studies [84,85,86]. When compared to a placebo, SGLT2 inhibitors can significantly avoid gaining body weight [87]. These conclusions have significant clinical inferences for Asians, as higher abdominal obesity contributes to insulin resistance, which leads to a risk of CVD. Dapagliflozin may demonstrate a dual function in improving glycemic control and lowering the risk of CVD in the Asian population. The practise of SGLT 2 inhibitors appears to be related to decreased levels of atherogenic, small, intense, low-density cholesterol in open-label Japanese cohorts [88]. In Chinese individuals with T2DM, the occurrence of chronic renal disorder and reduced hematocrit levels elevated the risk of severe cardiac outcomes. Elevated hematocrit levels in diabetic patients after SGLT2 inhibitor treatment may reverse kidney remodelling. During therapy with empagliflozin, elevated hematocrit levels were linked to a lower risk of cardiovascular death [89,90]. These findings imply that SGLT2 inhibitors may have the potential to decrease the cardiac burden in DM (Figure 3).

## 6. Conclusions

East Asians are at higher risk due to an increased prevalence of diabetes. The following factors contribute to the improved incidence of T2DM and the ethnic-specific character of East Asians, insulin secretion, rising BMI, stress, a proclivity for visceral fat, decreased pancreatic β-cell mass, diabetes onset at an early age, and cardiac events. SGLT2 inhibitors are novel antihyperglycemic agents with the possibility to progress in glycemic management while posing an incredibly low risk of hypoglycemia, insulin independence, and cardioprotection.

## Figures and Tables

**Table 1:** SGLT2 inhibitors studies on both mono and double therapy in East Asians.

Author; Region	Study Design and Population	Drug Therapy	Key Findings
Yang et al. China [54]	24-week, randomized, Phase 3, double-blind, placebo-controlled (*n* = 272)	Dapagliflozin	Significant reduction with Dapagliflozin HbA1c Fasting plasma glucose Body weight
Kawamori et al. Japanese [55]	52-week, randomized, double-blind, placebocontrolled study (*n* = 433)	Empagliflozin	Significant reduction with Empagliflozin HbA1c Fasting plasma glucose Body weight Systolic blood pressure
Kutoh et al. Japanese [56]	3-month, observational (*n* = 36)	Canagliflozin	Significant reduction with Canagliflozin HbA1c Free fatty acid Body weight insulin resistance
Osonoi et al. Japanese [57]	12-week, open label study (*n* = 20)	Canagliflozin	Significant reduction with Canagliflozin HbA1c Fasting plasma glucose Body weight
Furukawa et al. Japanese [58]	24-week, open label study (*n* = 104)	Dapagliflozin	Significant reduction with Dapagliflozin HbA1c Fasting plasma glucose Body weight low-density lipoprotein cholesterol
Han et al. Korean [59]	24-week, randomized, placebo-controlled, double-blind study (*n* = 104)	Ipragliflozin	Sustained reduction with Ipragliflozin HbA1c Fasting plasma glucose Body weight Fasting serum insulin
Chieh-Hsiang Lu et al. Korea and Taiwan [60]	24-week, multicenter, placebo-controlled, double-blind, parallel-group (*n* = 87)	Ipragliflozin	Significant reduction with Ipragliflozin HbA1 Fasting plasma glucose Body weight and waist circumference low-density lipoprotein cholesterol
